# Edible Yellow Mealworm-Derived Antidiabetic Peptides: Dual Modulation of α-Glucosidase and Dipeptidyl-Peptidase IV Inhibition Revealed by Integrated Proteomics, Bioassays, and Molecular Docking Analysis

**DOI:** 10.3390/foods15010096

**Published:** 2025-12-29

**Authors:** Yuying Zhu, Enning Zhou, Yingran Tang, Qiangqiang Li, Liming Wu

**Affiliations:** State Key Laboratory of Resource Insects, Institute of Apicultural Research, Chinese Academy of Agricultural Sciences, Beijing 100093, China

**Keywords:** yellow mealworm (*Tenebrio molitor*), antidiabetic peptides, α-glucosidase, dipeptidyl-peptidase IV, glucose consumption, molecular docking

## Abstract

Type 2 diabetes mellitus (T2DM) poses a critical global health burden, necessitating safer multi-target therapies. We pioneer the exploration of novel bioactive peptides from *Tenebrio molitor* larvae—an underexplored, sustainable, and edible insect protein—through proteomics-guided screening and bioassays. Six unique peptides (DK-7, WK-6, GR-7, FK-8, SK-6, and DK-8) demonstrated significant α-glucosidase and dipeptidyl-peptidase IV (DPP-IV) inhibitory effects, and significant glucose consumption enhancement in insulin-resistant HepG2 cells. Molecular docking revealed a binding topology where peptides interacted with α-glucosidase at its active sites (Glu271, Arg643, Arg647, Arg653, Tyr733, Lys765, and Glu767) and with DPP-IV at active residues (Phe357, Tyr547, Trp629, Asp729, and Gln731) through dual hydrogen-bond networks and hydrophobic interactions, establishing a novel inhibition mechanism. We wish to propose that insect-derived biopeptides have potential value as next-generation therapeutics, simultaneously advancing sustainable drug discovery and approximating functional food bioresources to biomedicine.

## 1. Introduction

Diabetes mellitus (DM) represents a persistent metabolic dysfunction marked by disruptions in blood glucose regulation, primarily resulting from insulin deficiency or insulin resistance (IR) [1]. It is closely linked to both microvascular and macrovascular complications, significantly elevating cardiovascular risk and posing a threat to patient health [2]. The World Health Organization (WHO) categorizes DM into three primary forms: type 1 diabetes mellitus (T1DM), type 2 diabetes mellitus (T2DM), and pregnancy-related gestational diabetes mellitus (GDM) [3]. Currently, DM represents a major global public health concern, affecting 537 million adults worldwide, with over 90% of cases classified as T2DM. Notably, the disease is increasingly manifesting at younger ages [4]. Therefore, targeted prevention strategies for T2DM are imperative to mitigate the overall burden of DM and address the rising prevalence of metabolic disorders in younger populations.

α-Glucosidase and dipeptidyl peptidase-IV (DPP-IV) are both key enzymes implicated in the pathophysiology of T2DM [5]. α-Glucosidase catalyzes the hydrolysis of dietary carbohydrates into absorbable monosaccharides, such as D-glucose, thereby contributing to postprandial hyperglycemia following intestinal absorption [6,7]. Key intestinal hormones such as glucagon-like peptide-1 (GLP-1) and glucose-dependent insulinotropic polypeptide (GIP) stimulate pancreatic insulin release in response to circulating glucose levels. However, these peptides are rapidly inactivated by DPP-IV, leading to reduced insulinotropic effects and impaired glycemic regulation [8]. Therefore, dual inhibition of α-glucosidase and DPP-IV represents a promising therapeutic strategy for improving glycemic control in T2DM [7].

Current clinical management of T2DM primarily relies on insulin therapy and oral hypoglycemic agents, such as α-glucosidase inhibitors (e.g., acarbose), biguanides (e.g., metformin), sulfonylureas (e.g., gliclazide), and DPP-IV inhibitors (e.g., sitagliptin). α-Glucosidase inhibitors reversibly inhibit α-glucosidase activity, thereby reducing oligosaccharide hydrolysis and monosaccharide absorption, which helps alleviate postprandial hyperglycemia [9,10]. DPP-IV inhibitors enhance GLP-1 activity, suppressing glucagon and stimulating insulin secretion. However, these agents face limitations: high production costs, adverse effects (e.g., hypoglycemia, gastrointestinal disturbances), and potential insulin resistance exacerbation with long-term insulin use [11,12]. Therefore, the development of safer and more effective inhibitors targeting both α-glucosidase and DPP-IV remains a crucial objective in diabetes treatment.

Bioactive peptides have attracted considerable attention owing to their multifunctional bioactivities, diverse origins, and favorable safety–stability profiles, demonstrating critical potential in food and medical applications. Recent studies have successfully isolated numerous hypoglycemic peptides from natural food sources. For instance, two Pu-erh tea-derived peptides, AS-9 and AR-9, were identified as potent α-glucosidase inhibitors with no observed hemolytic activity [13]. Additionally, two peptides (YGF and GMCC) derived from fermented coffee protein hydrolysates exhibited notable α-glucosidase inhibitory effects through interactions with catalytic residues and showed no cytotoxicity [7]. A tripeptide SGR derived from β-conglycinin was found to simultaneously inhibit α-glucosidase, enhance glucose metabolism in IR-HepG2 cells, and alleviate hyperglycemia in a T2DM zebrafish model [14]. Collectively, these findings underscore the therapeutic potential of naturally derived hypoglycemic peptides.

Recent studies have highlighted the screening and identification of hypoglycemic peptides derived from edible insects as a promising research area [15]. The yellow mealworm (*Tenebrio molitor*), a widely cultivated insect species, offers significant sustainability benefits, such as low rearing costs, minimal greenhouse gas emissions, and rapid reproduction rates, making it attractive to both the food and pharmaceutical industries. Moreover, the yellow mealworm has garnered attention owing to its rich nutritional profile, including high-quality protein, essential amino acids, and unsaturated fatty acids [16,17]. In 2021, the European Commission authorized dried mealworm larvae as a novel food, recognizing its developmental promise [18]. It exhibits therapeutic potential for Alzheimer’s, obesity, and osteoporosis, along with antibacterial and anti-inflammatory properties, supporting its functional food applications [19]. In traditional utilization of Indonesian medicine, the yellow mealworm was used as a method for hypoglycemic treatment [19]. Thus, it is of great research value to screen and identify the bioactive peptides with hypoglycemic effect from the yellow mealworm.

Molecular docking is a key in silico screening strategy, widely applied in high-throughput screening, drug design, and ligand–receptor interaction prediction (e.g., enzyme inhibitors) [20]. This study explores yellow mealworm larvae-derived bioactive peptides via integrated in vitro and in silico approaches to elucidate hypoglycemic mechanisms. Proteomics identified potential peptides, with PeptideRanker screening revealing six bioactive candidates for further study. In vitro assays demonstrated their excellent α-glucosidase and DPP-IV inhibition and enhanced glucose uptake in IR-HepG2 cells, suggesting their potential for IR mitigation. Molecular docking further elucidated their inhibitory mechanisms against α-glucosidase and DPP-IV. The findings provide theoretical and empirical support for developing novel hypoglycemic agents.

## 2. Materials and Methods

### 2.1. Reagents

HPLC-grade methanol, acetonitrile, and formic acid were sourced from Fisher Scientific (Pittsburgh, PA, USA). Enzymatic materials, such as α-glucosidase and substrate p-nitrophenol-β-D-galactoside (pNPG), came from Yuanye Biotechnology (Shanghai, China), with recombinant human insulin solution provided by Absin Biotechnology (Shanghai, China). Deionized water was prepared using Millipore’s Milli-Q purification system (Millipore, Burlington, MA, USA). Additional chemical reagents were purchased from Sangon Biotech (Shanghai, China).

### 2.2. Pretreatments of Yellow Mealworm Larvae

Commercially available yellow mealworm larvae could be procured directly. The rearing conditions are as follows: A density of up to 2 kg per square meter is advised, with the substrate layer not exceeding 2 cm in thickness in a growth chamber. The diet composition consists of 70% wheat bran, 15% cornmeal, and 15% oilseed cake, and fresh vegetable leaves or fruit peels may be supplemented periodically. The optimal temperature range for larval development is 13–32 °C, with a relative humidity maintained between 80% and 85%. Yellow mealworm larvae samples were subjected to freeze-drying and subsequently ground into a fine powder. The ratio of substrate to enzyme was 50:1. The powdered sample (10 g) was combined with trypsin enzyme (enzyme activity of 2500 U/mg, 0.2 g) in 10 mL of ultrapure water. This solution underwent pH adjustment to 8.0 through the mixed liquids of NaOH and NaHCO_3_, followed by thermal incubation at 37 °C for 4 h to promote proteolytic activity, and formic acid terminates enzymatic hydrolysis reactions. Following enzyme treatment, post-digestion samples were subjected to secondary lyophilization and cryopreserved at −80 °C to yield yellow mealworm enzymatic hydrolysates. Peptide extraction from yellow mealworm enzymatic hydrolysates was performed by the Insect Tissue Total Protein Extraction Kit (Invent Minute^TM^)(Eden Prairie, MN, USA), followed by purification via Millipore ZipTip C18 microcolumns, and quantification was performed using a Thermo Nanodrop2000 (Waltham, MA, USA) with subsequent normalization. Finally, after filtration through 0.22 μm filters, the samples underwent nLC-ESI-MS/MS detection and analysis as described by Meng et al. [21].

### 2.3. Identification of Bioactive Peptides from Yellow Mealworm Larvae

Chromatographic separation was achieved using an EASY-nLC1000 system (Waltham, MA, USA) equipped with a reversed-phase C18 column (75 μm internal diameter × 15 cm length, 3 μm particle size, and 100 Å pore size) interfaced with an LTQ-Orbitrap Elite mass spectrometer operating in positive electrospray ionization mode. The mobile phase consisted of two components: solution A (ultrapure water containing 0.1% formic acid) and solution B (acetonitrile with 0.1% formic acid). A multi-stage gradient protocol was implemented: initial 3% B (0–5 min), linear increase to 20% B (5–85 min), subsequent elevation to 30% B (85–105 min), and rapid ascent to 90% B (105–110 min), followed by isocratic maintenance at 90% B (110–120 min). A constant flow rate of 350 nL/min was maintained throughout the analytical run.

Mass spectrometry datasets were initially collected through Xcalibur 2.2 (Thermo Fisher Scientific, Waltham, MA, USA) and analyzed using Peaks DB 7.5 (Bioinformatics Solutions Inc., Waterloo, ON, Canada) for comprehensive characterization. Sequence alignment of identified peptides was performed against the *Tenebrio molitor* proteomic database sourced from the UniProt repository. Analytical parameters were established as follows: precursor ion mass accuracy threshold set at ±15 ppm; peptide mass error tolerance of ±0.05 Da; digestion enzyme specified as trypsin; permitted missed cleavage sites limited to 2; allowance for up to 3 variable post-translational modifications per peptide; and fixed modification applied as cysteine alkylation via iodoacetamide (C, +57.02 Da). Post-translational modifications included methionine oxidation (M, +15.99 Da). The decoy database approach maintained false discovery rates (FDR) below 1% threshold. Label-free quantitative analysis (LFQ) using the Peaks Q module calculated protein/peptide abundance ratios. Experimental design involved triplicate measurements per sample, with system-selected representative specimens for detailed examination. Analytical specifications encompassed ±0.5 s retention window alignment and a precursor mass tolerance of 15 ppm [21,22].

### 2.4. In Silico Screenings of Bioactive Peptides

The potential bioactive peptides from the yellow mealworm were initially predicted using PeptideRanker (http://distilldeep.ucd.ie/PeptideRanker/, accessed on 10 May 2024). Peptides with a bioactivity score of ≥0.7 were selected for further investigation. A higher score indicates a greater likelihood of bioactivity [23]. The bioactive peptides for subsequent assays were identified and, consequently, synthesized from the Sangon Biotech company (Shanghai, China), and the information about the synthesized bioactive peptides is shown in the Supplementary Materials in Figures S1–S6.

### 2.5. Inhibitory Effects of Bioactive Peptides on α-Glucosidase and DPP-IV

We refined the protocol proposed by Zhang et al. [24] to assess the in vitro inhibitory effects of yellow mealworm larvae bioactive peptides on α-glucosidase. Specifically, all samples and reagents were prepared and diluted in PBS (pH = 6.8). Bioactive peptide solutions were prepared in a gradient series spanning 1 to 10 mg/mL concentrations. Aliquots (10 μL) from each dilution were dispensed into separate wells of a 96-well microplate. These were combined with 100 μL phosphate-buffered saline (PBS) and 10 μL α-glucosidase enzyme solution (7 U/mL), followed by a 15 min incubation period at 37 °C. After adding 10 μL p-nitrophenyl-α-D-glucopyranoside (pNPG) substrate (2.5 mmol/L), the plates underwent an additional 30 min incubation under identical thermal conditions. Optical density measurements at 405 nm were subsequently obtained using a SpectraMax M5 microplate reader (Molecular Devices, Sunnyvale, CA, USA). Control experiments utilized 10 μL PBS in place of peptide solutions to establish baseline measurements. The inhibitory activity against α-glucosidase was quantified through computational analysis, as detailed in Equation (1), and the IC_50_ values can also be calculated based on the inhibition rates of each bioactive peptide:

(1) α-Glucosidase inhibition rate (%) = [(A_Blank_ − A_Sample_)/A_Blank_] × 100

where A represents the absorbance value.

Subsequently, the screening assay was conducted using a commercial kit (Abcam, Cambridge, UK) following the protocols provided by the manufacturer. The half-maximal inhibitory concentration (IC_50_) was determined through analysis of DPP-IV inhibition rates, applying the formula outlined in Equation (2):

(2) DPP-IV inhibition rate (%) = [(A_Blank_ − A_Sample_)/A_Blank_] × 100

where A represents the absorbance value.

### 2.6. Establishment of Cell Cultures and the IR-HepG2 Cell Model

HepG2 human hepatocellular carcinoma cells utilized in this study were generously provided by Prof. Fuliang Hu from Zhejiang University’s College of Animal Science (China). Cellular maintenance involved cultivation in HyClone’s high-glucose DMEM medium supplemented with 10% heat-inactivated fetal bovine serum (Gibco, Waltham, MA, USA) and antibiotic agents (100 U/mL penicillin plus 100 μg/mL streptomycin). Cellular propagation was conducted under standard culture conditions (37 °C, 5% CO_2_ humidified atmosphere), following established protocols [25].

In order to systematically evaluate whether the bioactive peptides from yellow mealworm can effectively decrease the levels of glucose consumption, the IR-HepG2 cell model was established for validation. The experimental protocol referenced the method described by Ma et al. [26]: Firstly, the HepG2 cells were digested using a 0.25% (mass fraction) trypsin solution and seeded into 96-well plates until the cells adhered. Subsequently, the insulin solutions with different concentration gradients needed to be prepared. We set 6 various insulin concentration gradients: 1 × 10^−4^, 1 × 10^−5^, 1 × 10^−6^, 1 × 10^−7^, 1 × 10^−8^, and 1 × 10^−9^ mol/L, and these insulin solutions were diluted, prepared by DMEM medium, and added to the wells for 24 h incubation. Following the incubation, 10 μL of Cell-Counting Kit-8 solution (CCK-8) was added, and the plates were gently shaken to ensure uniform mixing before being incubated again at 37 °C under 5% CO_2_ for another 1 h. Finally, the cell viability under different insulin concentrations was detected by measuring absorbance at 450 nm using a microplate reader and calculated according to Equation (2) to identify the optimal insulin concentration for the IR-HepG2 cell model.

Meanwhile, the determination of the optimal insulin action time was refined based on the methodology proposed by Hu et al. [27]. The procedures for enzyme digestion and cell seeding are identical to those detailed in the previous section. Following cell adherence in 24-well plates, the optimal insulin concentration was administered and cultured for varying durations (12 h, 24 h, 36 h, and 48 h). Subsequently, the glucose consumption in the supernatant of cells was quantified using a D-glucose detection kit (Megazyme, Bray, Ireland). Finally, the glucose consumption was calculated according to Equation (3) to identify the optimal action time for the IR-HepG2 cell model. All experiments included blank groups consisting of DMEM high-glucose medium devoid of insulin:

(3) Cell viability (%) = (A_Sample_/A_Blank_) × 100%

(4) Glucose consumption (mmol/L) = 4 − (A_Sample/Blank_/A_Standard_) × 4

where A represents the absorbance value.

### 2.7. Effects of Bioactive Peptides on Cell Viability

To validate whether the bioactive peptides exert toxic effects on cells, the impact of peptide concentrations on cell viability should also be assessed. The specifics are exhibited below: Following cell adhesion, first, the bioactive peptide samples were formulated in DMEM medium at concentration gradients of 50, 100, 200, 400, and 800 μg/mL, and then they were administered to the experimental groups and incubated for 24 h. After the treatment period, 10 μL of CCK-8 solution was added, and these 96-well plates were gently shaken and incubated again at 37 °C under 5% CO_2_ for another 1 h. In the end, the calculation methodology for evaluating the cell viability of bioactive peptides is described in Equation (2) in Section 2.6 to assess the effects of bioactive peptides on cell viability. All blank groups were established using DMEM high-glucose medium devoid of insulin.

### 2.8. Effects of Bioactive Peptides on Glucose Consumption in IR-HepG2 Cells

We adapted the method described by Zang et al. [28] to examine the glucose consumption in the IR-HepG2 cell model. Specifically, after the adherence of HepG2 cells in 24-well plates, each plate was assigned to three groups that were named the blank group, insulin group, and diverse peptide group. Firstly, the fresh phenol-red-free DMEM medium was utilized to prepare insulin solutions at concentrations of 1 × 10^−6^ and 2 × 10^−6^ mol/L, coupled with bioactive peptides solutions at concentrations of 200, 400, and 800 μg/mL. Next, the distinct experimental groups required the addition of specific reagents tailored to their respective treatment conditions: Blank group was phenol-red-free high-glucose DMEM, insulin group received 1 × 10^−6^ mol/L insulin solution, and peptide group received a rate of 1:1 mixture of high-concentration insulin solution (2 × 10^−6^ mol/L) and bioactive peptide solutions (200, 400, and 800 μg/mL), respectively. All groups were incubated for 24 h under standard conditions. Finally, followed by taking the supernatant of each group, the glucose content was quantified using a D-glucose detection kit (Megazyme, Ireland), and the glucose consumption for each group was calculated according to Formula (3) (Section 2.6). Consequently, the abilities of bioactive peptides for mitigating the IR of HepG2 cells could be validated successfully.

### 2.9. Molecular Docking Analysis

Using the PyMOL 1.8 molecular visualization software, protein structures were prepared by removing all ligands and water molecules from the X-ray crystallographic data of human α-glucosidase (PDB ID: 2QMJ) and DPP-IV enzyme (PDB ID: 1WCY), which were obtained from the Protein Data Bank (PDB) repository. Subsequently, the 2D structures of 6 bioactive peptide segments were constructed using ChemDraw 2D 19.0 software (PerkinElmer, Waltham, MA, USA) and converted into 3D structures via ChemDraw 3D 19.0 software (PerkinElmer, Waltham, MA, USA). The Molecular Mechanics 2 (MM2) method was employed to optimize the conformations of these peptide segments.

For molecular docking, a cubic grid box with dimensions of 45.0 Å × 47.5 Å × 38.8 Å and a grid spacing of 0.375 Å was defined, and the centers of α-glucosidase and DPP-IV located at coordinates (X = −28.6, Y = 8.975, Z = −15.921 and X = 74.7, Y = 56.6, Z = 36.6, respectively) were set for further analysis. Molecular docking studies were conducted with AutoDock Vina 1.2.3 software [29], where all compounds underwent ten separate docking trials. The most favorable binding energy conformation from each series was subsequently chosen for detailed interaction analysis. Hydrogen bonds and hydrophobic contacts were mapped through the Protein-Ligand Interaction Profiler platform (https://plip-tool.biotec.tu-dresden.de/plip-web/plip/index, accessed on 20 May 2024). Finally, the resulting complex structures were visualized using PyMOL 1.8 software, thereby obtaining the consequences of molecular docking [30].

### 2.10. Statistics

Statistical evaluation employed Student’s *t*-test and one-way ANOVA via SPSS 21.0, with significance thresholds established at *p* < 0.05.

## 3. Results and Discussion

### 3.1. In Silico Screening of Bioactive Peptides from Yellow Mealworm Larvae

Peptides in yellow mealworm larvae were systematically identified and characterized using proteomics, involving high-throughput mass spectrometry and bioinformatics analysis. Sequence alignment was performed against the *Tenebrio molitor* protein database downloaded from Uniprot. PeptideRanker is an in silico prediction platform designed for the screening of unidentified bioactive peptides based on functional category-specific features. The score system ranges from 0 to 1, and the scores closer to 1 indicate a higher probability of bioactivity [31]. Based on the previous content, we employed PeptideRanker in conjunction with bioinformatics techniques to perform an initial screening of bioactive peptides from the yellow mealworm with a score ≥ 0.7. Prediction results were shown in Table 1. 6 peptides with a ranker score > 0.7 exhibiting a high possibility of biological activity were identified for further investigation, including DYGPPFK (DK-7), WSPDPK (WK-6), GMDFQPR (GR-7), FNPFDLTK (FK-8), SLFLPK (SK-6), and DPFDALPK (DK-8).

### 3.2. Inhibitory Effects of Six Bioactive Peptides on α-Glucosidase and DPP-IV

α-Glucosidase is a critical enzyme involved in energy metabolism and the maintenance of normal physiological functions [32]. This enzymatic process facilitates the cleavage of α-1,4 glycosidic linkages at non-reducing termini in oligosaccharides or disaccharides, liberating glucose molecules that subsequently influence metabolic regulation in T2DM [5]. Dipeptidyl peptidase-IV (DPP-IV) serves as an imperative therapeutic target for T2DM treatment due to its role in degrading incretin hormones [33]. This investigation assessed the inhibitory potential of six candidate bioactive peptides targeting α-glucosidase and DPP-IV enzymes. Experimental data (Table 2) revealed measurable α-glucosidase inhibition across all tested peptides, with half-maximal inhibitory concentrations falling between 5.58 and 11.06 mg/mL. However, only three peptides showed inhibitory effects against DPP-IV. Among them, GR-7 demonstrated the highest inhibitory potency against DPP-IV, with an IC_50_ value of 0.64 mg/mL, followed by DK-8 (IC_50_ = 7.93 mg/mL) and WK-6 (IC_50_ = 9.20 mg/mL). Similar findings have been reported in previous studies: The AR-9 peptide derived from ripened Pu-erh tea exhibited significant α-glucosidase inhibitory activity, with an IC_50_ value of 3.94 mg/mL [13]. Notably, previous research identified a salmon skin gelatin-derived peptide demonstrating potent DPP-IV inhibition, recording an IC_50_ of 1.35 mg/mL [34]. Therefore, our findings indicate that the GR-7 peptide possesses potent hypoglycemic activity.

### 3.3. Effects of Six Bioactive Peptides on Glucose Consumption in IR-HepG2 Cell Model

**Figure 1:**
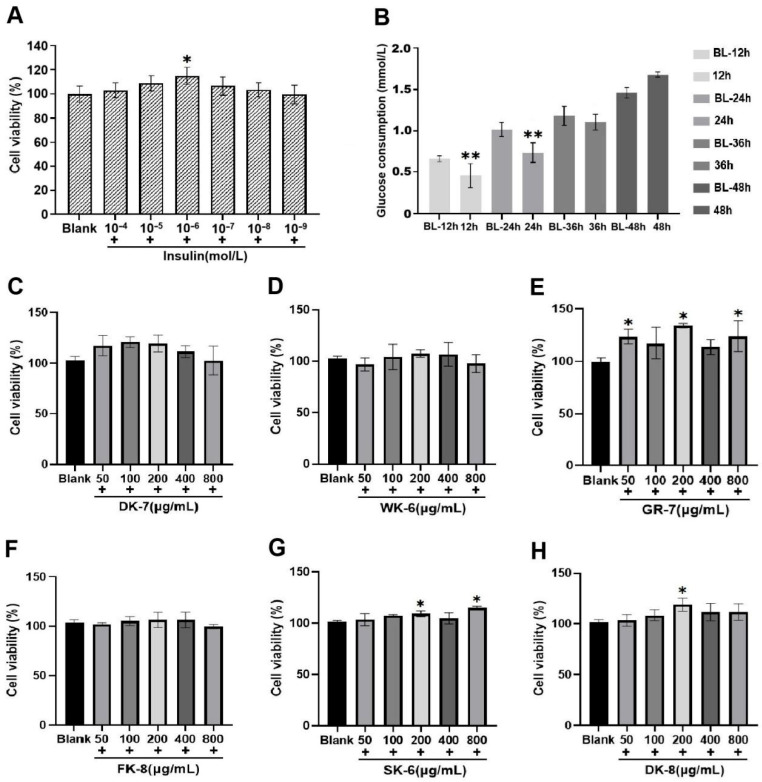
(**A**) Effect of different insulin concentrations on HepG2 cell viability. (**B**) Effect of different exposure durations on glucose consumption in the insulin resistance (IR) HepG2 cell model. (**C**–**H**) Effects of 6 bioactive peptides from yellow mealworm larvae (*Tenebrio molitor*) at different concentrations on HepG2 cell viability. Asterisks (*) and (**) indicate statistically significant differences (*p* < 0.05) and (*p* < 0.01), respectively.

Insulin resistance (IR) represents a defining characteristic of type 2 diabetes pathophysiology [35]. To evaluate the hypoglycemic activity of six bioactive peptides, we established an IR-HepG2 cell model. First, we determined the optimal insulin concentration for model induction. Cell viability assays revealed a biphasic response: viability initially increased and then decreased as insulin concentrations ranged from 1 × 10^−9^ to 1 × 10^−4^ mol/L. Crucially, at an insulin concentration of 1 × 10^−6^ mol/L, cell viability analysis indicated maximal viability enhancement at 1 × 10^−6^ mol/L insulin concentration, showing statistically significant elevation versus control samples (*p* < 0.05, Figure 1A). This optimized concentration was subsequently employed for model development. Next, we optimized the insulin exposure duration. HepG2 cells were treated with 1 × 10^−6^ mol/L insulin for 12 h, 24 h, 36 h, or 48 h, and glucose consumption was measured at each time point. The results showed that after 24 h of insulin treatment, glucose consumption was significantly lower compared to the corresponding blank group (treated for 24 h without insulin, *p* < 0.01) (Figure 1B). The insulin-resistant cellular model was successfully developed through 24 h exposure of HepG2 cells to 1 × 10^−6^ mol/L insulin, establishing a stable platform for subsequent experimental investigations.

Furthermore, to identify the optimal concentration range of the bioactive peptides, we established a series of concentration gradients (50, 100, 200, 400, and 800 μg/mL) and assessed their effects on HepG2 cell viability. As illustrated in Figure 1C–H, cell viability remained above 95% within the concentration range of 100 to 400 μg/mL, indicating that these concentrations were non-toxic to HepG2 cells. Notably, at certain concentrations within this safe range (e.g., for GR-7, SK-6, and DK-8), cell viability was even significantly higher than the blank group (*p* < 0.05), suggesting a potential proliferative effect. Based on the non-toxicity observed across 100–400 μg/mL and the potential bioactivity indicated by the increased viability for some peptides, the concentrations of 100, 200, and 400 μg/mL were selected for subsequent evaluation of glucose consumption in IR-HepG2 cells.

**Figure 2:**
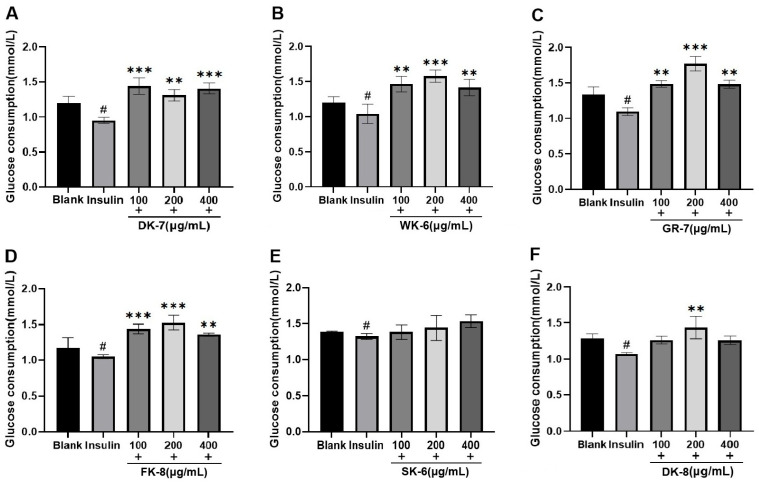
The effects of 6 bioactive peptides from yellow mealworm larvae (*Tenebrio molitor*) on glucose consumption in the IR-HepG2 cell model. (**A**) DK-7, (**B**) WK-6, (**C**) GR-7, (**D**) FK-8, (**E**) SK-6, and (**F**) DK-8. Symbols indicate statistical significance: # *p* < 0.05 vs. untreated control group; ** *p* < 0.01 and *** *p* < 0.001 vs. insulin-treated group.

Diabetes mellitus (DM) profoundly disrupts glucose homeostasis. To evaluate the effects of six bioactive peptides on glucose metabolism, we employed the HepG2 cell line—a well-established model that closely mimics human hepatocytes [36]—focusing specifically on glucose uptake. Within the IR-HepG2 cell model, enhanced glucose consumption directly reduces residual glucose levels in the system, thereby contributing to blood glucose lowering. As shown in Figure 2, insulin-treated groups exhibited significantly reduced glucose consumption compared to blank groups, validating successful IR induction. All six bioactive peptides could increase the glucose consumption, significantly surpassing levels observed in both blank and insulin-treated groups. Notably, for peptides WK-6, GR-7, FK-8, and DK-8, glucose consumption exhibited a distinct biphasic response: it increased initially with peptide concentration but peaked at 200 μg/mL before declining at the highest concentration (400 μg/mL). These increases at peak concentrations (200 μg/mL) were statistically significant compared to the insulin-treated groups (*p* < 0.001 or *p* < 0.01, Figure 2B–D,F, respectively).

Our findings align with previous research demonstrating that bioactive peptides are capable of enhancing glucose consumption in IR models. For instance, Wang et al. [37] identified the pentapeptide LPLLR from walnut protein hydrolysate, which enhanced glucose consumption in IR-HepG2 cells while also inhibiting α-glucosidase. Similarly, Ding et al. [38] isolated and characterized the novel peptide YPLPR from distillers’ grains, and this peptide also demonstrated a dual function in vitro: it stimulated glucose consumption in IR-HepG2 cells and concurrently exhibited inhibitory activity against α-glucosidase. After employing a comparable methodology, we have identified six bioactive peptides derived from the yellow mealworm (*Tenebrio molitor*) that significantly enhance glucose uptake in IR-HepG2 cells, offering potential for blood glucose regulation.

### 3.4. Molecular Docking Reveals High-Affinity Binding of Six Bioactive Peptides to the Target Enzymes α-Glucosidase and DPP-IV

To investigate the binding mechanisms of six bioactive peptides with the hypoglycemia-related enzymes α-glucosidase and DPP-IV, computational molecular docking techniques were employed. The visualization and analysis of docking outcomes were conducted through PyMOL v2.3.1 software, employing its advanced molecular graphics capabilities to elucidate molecular interactions. Guided by established biochemical principles, enhanced enzyme inhibition is consistently associated with stronger ligand–receptor binding, which is reflected in more favorable (more negative) calculated binding free energies (ΔG) [39]. All peptides exhibited substantial binding affinity to α-glucosidase (Table S1). Notably, WK-6 demonstrated the strongest interaction (ΔG = −8.2 kcal/mol), paralleling the positive control WLRL (−8.2 kcal/mol), a potent soy-derived inhibitor [40]. Four peptides (WK-6, SK-6, GR-7, and FK-8) achieved ΔG ≤ −8.0 kcal/mol, signifying high inhibitory potential—surpassing DK-7 and DK-8 (−8.0 ≤ ΔG ≤ −7.0 kcal/mol). For DPP-IV (Table S2), GR-7 showed exceptional affinity (ΔG = −9.0 kcal/mol), markedly lower than WLRL (−7.6 kcal/mol). Four peptides (GR-7, DK-7, WK-6, and FK-8) exhibited ΔG ≤ −8.0 kcal/mol, indicating robust binding, while DK-8 showed moderate affinity (ΔG = −6.8 kcal/mol).

**Figure 3:**
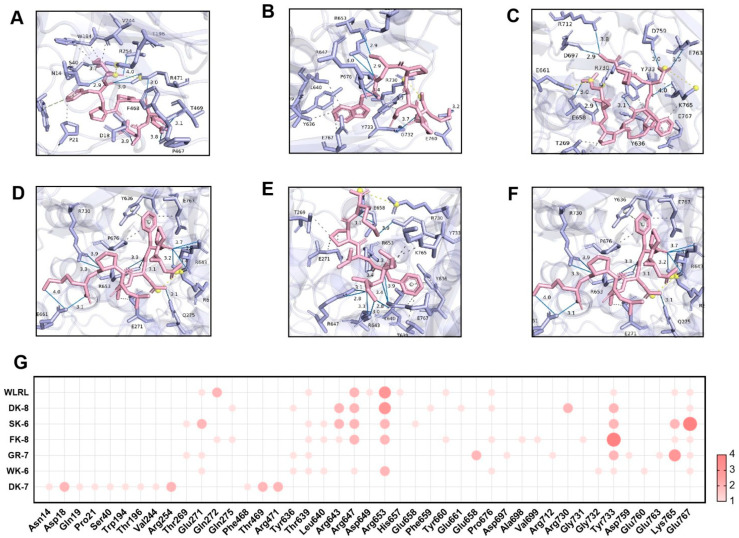
Molecular docking of 6 bioactive peptides with α-glucosidase (PDB ID: 2QMJ). (**A**–**F**) 3D diagrams depicting hydrogen bonds and hydrophobic interactions between the peptides and α-glucosidase amino acid residues: (**A**) DK-7, (**B**) WK-6, (**C**) GR-7, (**D**) FK-8, (**E**) SK-6, and (**F**) DK-8. (**G**) Docking site statistics. Bubble size and color intensity correspond to the number of interacting amino acid residues per peptide.

**Figure 4:**
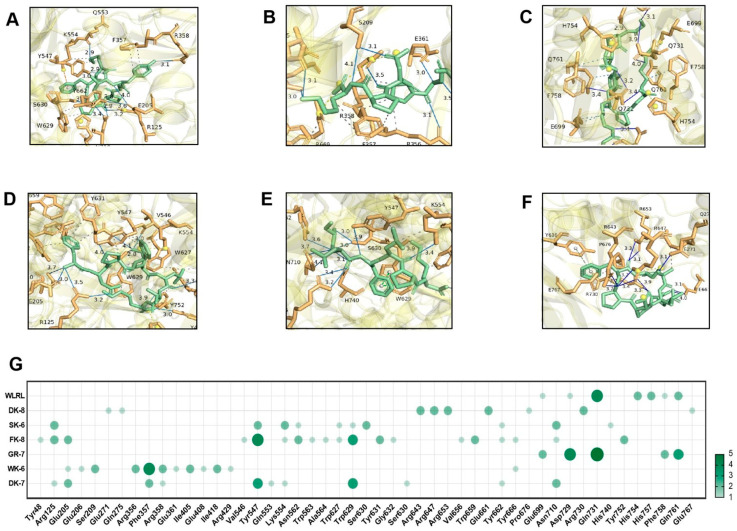
Molecular docking of 6 bioactive peptides with DPP-IV (PDB ID:1WCY). (**A**–**F**) 3D diagrams depicting hydrogen bonds and hydrophobic interactions between the peptides and α-glucosidase amino acid residues: (**A**) DK-7, (**B**) WK-6, (**C**) GR-7, (**D**) FK-8, (**E**) SK-6, and (**F**) DK-8. (**G**) Docking site statistics. Bubble size and color intensity correspond to the number of interacting amino acid residues per peptide.

Hydrogen bonding and hydrophobic interactions are critical determinants of ligand–receptor binding. Specifically, in the molecular docking between ligands and α-glucosidase or DPP-IV, the formation and stability of the complex are governed by the number and spatial distance of hydrogen bonds. These bonds enable the ligand to bind directly to key amino acid residues within the enzyme’s active site, thereby inhibiting α-glucosidase and DPP-IV activity by sterically blocking substrate access to the catalytic pocket [38]. Detailed 3D structures revealed hydrogen bonds (H-bonds) and hydrophobic interactions between peptides and catalytic pockets. H-bonds—the dominant interaction mechanism [41]—were extensively observed. For α-Glucosidase, peptides formed 7–13 H-bonds with 6–8 residues (Figure 3A–F) and 4–9 hydrophobic interactions with 4–9 residues (Table S1). For DPP-IV, interactions ranged from 7 to 12 H-bonds across 3–10 residues (Figure 4A–F) and 3–18 hydrophobic interactions with 3–13 residues (Table S2). Critically, average H-bond distances (3.2–3.4 Å) were below the 3.5 Å stability threshold [42], confirming complex stability. Moreover, for α-Glucosidase, residues Glu271, Arg643, Arg647, Arg653, Tyr733, Lys765, and Glu767 comprise the catalytic domain (358–720 aa) [43]. Strikingly, all peptides engaged conserved functional residues (Arg, Tyr)—validating target specificity. Five peptides additionally bound Glu767 (Figure 3G), a site critical for substrate recognition. This residue-level convergence suggests a shared inhibitory mechanism distinct from prior reports emphasizing Asp/Trp interactions [41,44]. For DPP-IV, binding centered on residues Phe357, Tyr547, Trp629, Asp729, and Gln731 (Figure 4G). Tyr547—a signature residue of the charged S1 pocket [33]—was consistently targeted, highlighting electrostatic complementarity as a driver of selectivity.

These structural insights directly elucidate experimental observations. Potent α-glucosidase inhibition correlates with deep catalytic pocket penetration (ΔG ≤ −8.0 kcal/mol) and multi-residue H-bond networks, enhanced by the newly identified Glu767 anchor. GR-7’s exceptional DPP-IV inhibition aligns with its ultra-low ΔG (−9.0 kcal/mol) and efficient binding (nine H-bonds across three residues). Critically, peptide-enhanced glucose consumption in IR-HepG2 cells (Figure 2) validates these in silico predictions, confirming *Tenebrio molitor* peptides as bona fide dual-target hypoglycemic agents.

While α-glucosidase and DPP-IV do not directly modulate insulin signaling, their indirect roles in glucose homeostasis are well-established. α-Glucosidase governs postprandial glycemia by catalyzing carbohydrate hydrolysis, directly impacting glucose flux. DPP-IV degrades incretins (e.g., GLP-1), attenuating insulin secretion [45]. Consequently, these bioactive peptides could ameliorate IR through dual mechanisms: (1) stabilizing glycemic variability via α-glucosidase inhibition to attenuate postprandial glucose excursions; and (2) preserving GLP-1 bioavailability via DPP-IV inhibition to potentiate insulinotropic signaling [5]. This synergistic action—simultaneously targeting glucose supply (α-glucosidase) and insulin efficacy (DPP-IV/GLP-1)—represents a significant advance over single-target approaches for managing insulin resistance [46].

## 4. Conclusions

In summary, compared with traditional hypoglycemic drugs, the core advantages and novelty of novel hypoglycemic bioactive peptides lie in the fundamental breakthroughs achieved in both the deepening and expansion of mechanisms of action and the key pharmaceutical properties. Structurally, novel hypoglycemic bioactive peptides are fundamentally characterized by their macromolecular nature, defined by specific amino acid sequences, and their complex three-dimensional conformations. This structural foundation confers upon them highly specific and high-affinity target recognition capabilities, enabling the rational integration of multiple mechanisms of action alongside glucose regulatory functions. Consequently, this allows for potent blood glucose-lowering efficacy while significantly reducing the risk of hypoglycemia. Mechanistically, the bioactive peptides transcend the single-target or incretin-based pathways of conventional drugs, demonstrating characteristics of multi-target, intelligent, and systemically coordinated regulation. They not only lower blood glucose safely but also show potential for addressing pathological root causes, such as improving insulin resistance and restoring islet function, representing a therapeutic paradigm shift from “glucose management” to “metabolic intervention and repair”. In addition, the bioactive peptides have also successfully overcome the classic challenges of low oral bioavailability and short half-life typical of peptide drugs. This progress lays a crucial foundation for developing oral and ultra-long-acting formulations, marking the emergence of a new paradigm in diabetes therapy.

Our study systematically elucidated the hypoglycemic mechanism and application potential of yellow mealworm larvae bioactive peptides through integrated in vitro enzymatic inhibition assays, cellular model validation, and in silico simulations. Six identified peptides exhibited potent α-glucosidase and DPP-IV inhibitory activity in vitro and stimulatory effects on glucose consumption in the IR-HepG2 cell model. Molecular docking revealed their inhibition mechanism involved hydrogen-bond networks and hydrophobic interactions at the active sites of α-glucosidase and DPP-IV. Notably, we innovatively established a multi-dimensional validation strategy (in vitro–cellular–in silico), providing novel candidates for insect-derived hypoglycemic peptides. Future work will focus on in vivo hypoglycemic efficacy and pharmacokinetics in diabetic animal models, and the development of nano-delivery systems for peptide-based functional foods. These investigations aim to address limitations of conventional hypoglycemic agents and provide theoretical foundations for developing safe and efficient diabetes interventions.

## Figures and Tables

**Table 1:** The identification of active peptides from the enzymatic hydrolysates of yellow mealworm larvae analyzed by Easy-nLC1000-LTQ-Orbitrap Elite mass spectrometer (Waltham, MA, USA).

Peptide Sequence	Abbreviation	Retention Time (min)	Mass (Da)	*m*/*z*	Peptide Ranker Score ^a^
DYGPPFK	DK-7	33.64	822.3912	412.2027	0.8825
WSPDPK	WK-6	10.80	728.3493	365.1817	0.8460
GMDFQPR	GR-7	21.99	849.3803	425.6973	0.8088
FNPFDLTK	FK-8	53.35	980.4967	491.2564	0.7960
SLFLPK	SK-6	31.50	703.4268	352.7203	0.7952
DPFDALPK	DK-8	44.05	901.4545	451.7343	0.7904

^a^ The potential bioactive peptides from yellow mealworm were initially predicted using PeptideRanker (http://distilldeep.ucd.ie/PeptideRanker/, accessed on 10 May 2024). A score of ≥0.7 indicates a high possibility of biological activity.

**Table 2:** The inhibitory effects of bioactive peptides from yellow mealworm larvae on α-glucosidase and DPP-IV.

Enzymes	Peptides	Standard Curves	R^2^	Linearity Ranges (mg/mL)	IC_50_ (mg/mL)
α-Glucosidase	DK-7	Y = 9.93 × 10^−5^ × X − 0.0722	0.9995	1–10	5.76
	WK-6	Y = 7.39 × 10^−5^ × X − 0.0782	0.9930	1–10	7.82
	GR-7	Y = 8.46 × 10^−5^ × X − 0.0356	0.9991	1–10	6.17
	FK-8	Y = 0.0458 × X − 0.0064	0.9949	1–12	11.06
	SK-6	Y = 5.27 × 10^−5^ × X − 0.0340	0.9953	1–10	10.14
	DK-8	Y = 1.03 × 10^−4^ × X − 0.0977	0.9968	1–10	5.58
DPP-IV	DK-7	Y = 2 × 10^−5^ × X + 0.0297	0.9927	1–5	-
	WK-6	Y = 3 × 10^−5^ × X + 0.2241	0.9928	1–10	9.20
	GR-7	Y = 8 × 10^−4^ × X − 0.0127	0.9983	0.1-1	0.64
	FK-8	Y = 1 × 10^−5^ × X + 0.0037	0.9959	1–10	-
	SK-6	Y = 1 × 10^−5^ × X + 0.0142	0.9990	1–10	-
	DK-8	Y = 5 × 10^−5^ × X + 0.1033	0.9925	1–10	7.93

## Data Availability

The original contributions presented in the study are included in the article/Supplementary Materials. Further inquiries can be directed to the corresponding authors.

## Supplementary Materials

The following supporting information can be downloaded at: https://www.mdpi.com/article/10.3390/foods15010096/s1, Figure S1: The chromatogram and the purity of the bioactive peptide sample DYGPPFK; Figure S2: The chromatogram and the purity of the bioactive peptide sample WSPDPK; Figure S3: The chromatogram and the purity of the bioactive peptide sample GMDFQPR; Figure S4: The chromatogram and the purity of the bioactive peptide sample FNPFDLTK; Figure S5: The chromatogram and the purity of the bioactive peptide sample SLFLPK; Figure S6: The chromatogram and the purity of the bioactive peptide sample DPFDALPK; Table S1: The molecular docking results of bioactive peptides from yellow mealworm larvae with α-glucosidase; Table S2: The molecular docking results of bioactive peptides from yellow mealworm larvae with DPP-Ⅳ.
