# Supplementary material for: Edible Yellow Mealworm-Derived Antidiabetic Peptides: Dual Modulation of α-Glucosidase and Dipeptidyl-Peptidase IV Inhibition Revealed by Integrated Proteomics, Bioassays, and Molecular Docking Analysis
Source: Foods. 2025 Dec 29;15(1):96. doi: 10.3390/foods15010096 (PMC12785325; doi:10.3390/foods15010096)
Supplement: Supplementary file 1 [file foods-15-00096-s001.zip › foods-4026889-supplementary.pdf]

Supplementary Materials

Sample Information

Name : P36697-1
Sequence : DYGPPFK
Modification : N/A
Lot.No : P36697-1-25040201

Pump A : 0.1%trifluoroacetic in 100%water
Pump B : 0.1%trifluoroacetic in 100%acetonitrile
Total Flow : 1.0ml/min
Wavelength : 214nm
Analytical column type : SHIMADZU Inertsil ODS-SP(4.6*250MM*5UM)
Dissolution method : 0.5mg sample dissolved to 0.5mL by 10%ACN and 90%H2O
Acquisition Time : 2025/04/07 09:49:49
Inj.Volume : 30ul

Time	Module	Action	Value
0.01	Pumps	B.Conc	17
20.00	Pumps	B.Conc	37

Chromatogram

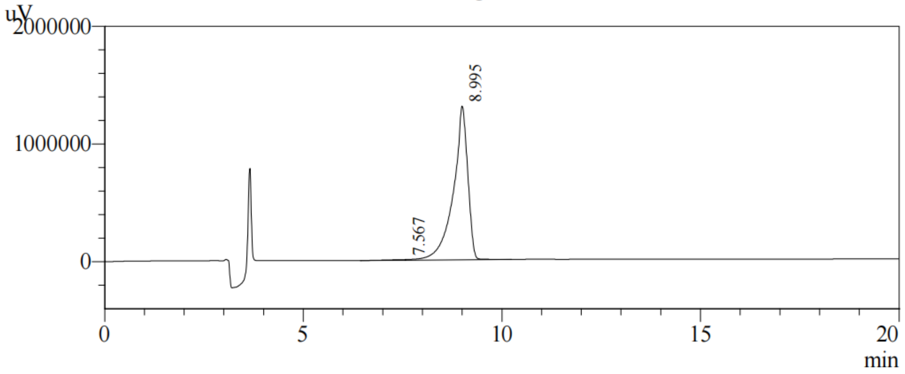

1 Det.A Ch1 / 214nm

PeakTable

Detector A Ch1 214nm

Peak#	Ret. Time	Area	Height	Area %	Height %
1	7.567	178298	5575	0.537	0.425
2	8.995	33037980	1306968	99.463	99.575
Total		33216278	1312543	100.000	100.000

Figure S1. The chromatogram and the purity of the bioactive peptide sample DYGPPFK.

Sample Information

Name	:	P36697-2
Sequence	:	WSPDPK
Modification	:	N/A
Lot.No	:	P36697-2-25040201
Pump A	:	0.1%trifluoroacetic in 100%water
Pump B	:	0.1%trifluoroacetic in 100%acetonitrile
Total Flow	:	1.0ml/min
Wavelength	:	214nm
Analytical column type	:	SHIMADZU shim-pack GIST C18(4.6*250MM*5UM)
Dissolution method	:	0.5mg sample dissolved to 0.5mL by 10%ACN and 90%H2O
Acquisition Time	:	2025/04/16 15:18:56
Inj.Volume	:	30ul

Time	Module	Action	Value
0.01	Pumps	B.Conc	13
20.00	Pumps	B.Conc	33

Chromatogram

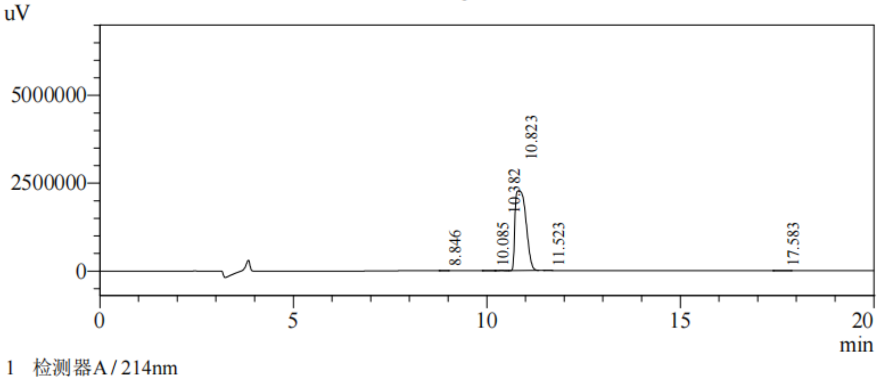

1 检测器A / 214nm

PeakTable

检测器A 214nm

Peak#	Ret. Time	Area	Height	Area %	Height %
1	8.846	14762	1864	0.033	0.082
2	10.085	13899	1563	0.031	0.069
3	10.382	35960	3998	0.081	0.175
4	10.823	44306564	2272628	99.829	99.625
5	11.523	5346	635	0.012	0.028
6	17.583	6089	499	0.014	0.022
总计		44382620	2281189	100.000	100.000

Figure S2. The chromatogram and the purity of the bioactive peptide sample WSPDPK.

Sample Information

Name	:	P36697-3
Sequence	:	GMDFQPR
Modification	:	N/A
Lot.No	:	P36697-3-25040201
Pump A	:	0.1%trifluoroacetic in 100%water
Pump B	:	0.1%trifluoroacetic in 100%acetonitrile
Total Flow	:	1.0ml/min
Wavelength	:	214nm
Analytical column type	:	SHIMADZU Inertsil ODS-SP(4.6*250MM*5UM)
Dissolution method	:	0.5mg sample dissolved to 0.5mL by 10%ACN and 90%H2O
Acquisition Time	:	2025/04/07 09:40:56
Inj.Volume	:	30ul

Time	Module	Action	Value
0.01	Pumps	B.Conc	15
20.00	Pumps	B.Conc	35

Chromatogram

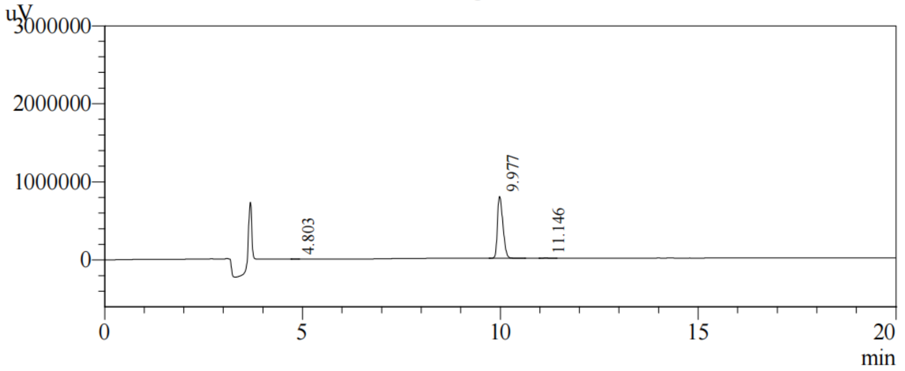

1 Det.A Ch1 / 214nm

PeakTable

Detector A Ch1 214nm

Peak#	Ret. Time	Area	Height	Area %	Height %
1	4.803	7635	1226	0.101	0.154
2	9.977	7563034	790627	99.558	99.439
3	11.146	25919	3232	0.341	0.407
Total		7596588	795086	100.000	100.000

Figure S3. The chromatogram and the purity of the bioactive peptide sample GMDFQPR.

Sample Information

Name	:	P36697-4
Sequence	:	FNPFDLTK
Modification	:	N/A
Lot.No	:	P36697-4-25040201
Pump A	:	0.1%trifluoroacetic in 100%water
Pump B	:	0.1%trifluoroacetic in 100%acetonrtrile
Total Flow	:	1.0ml/min
Wavelength	:	214nm
Analytical column type	:	SHIMADZU shim-pack GIST(4.6*250MM*5UM)
Dissolution method	:	0.1mg sample dissolved to 0.5mL by 10%HCOOH 20%ACN and 70%H2O
Acquisition Time	:	2025/04/08 12:24:12
Inj.Volume	:	30ul

Time	Module	Action	Value
0.01	Pumps	B.Conc	27
20.00	Pumps	B.Conc	47

Chromatogram

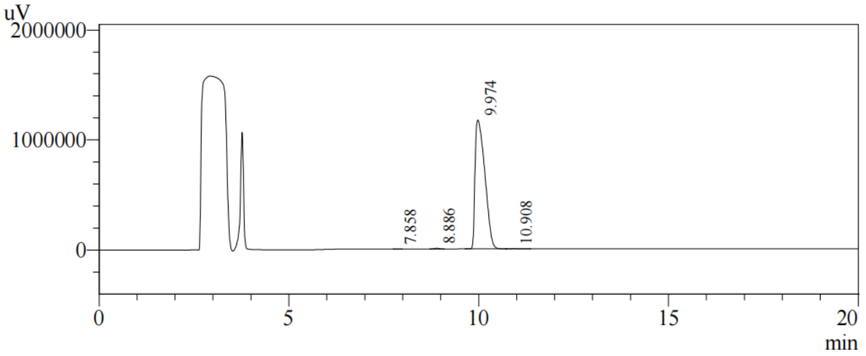

1 Det.A Ch1 / 214nm

PeakTable

Detector A Ch1 214nm

Peak#	Ret. Time	Area	Height	Area %	Height %
1	7.858	5259	666	0.025	0.057
2	8.886	58132	5942	0.277	0.504
3	9.974	20888504	1169016	99.587	99.245
4	10.908	23142	2285	0.110	0.194
Total		20975037	1177910	100.000	100.000

Figure S4. The chromatogram and the purity of the bioactive peptide sample FNPFDLTK.

Sample Information

Name : P36697-5
Sequence : SLFLPK
Modification : N/A
Lot.No : P36697-5-25040201
Pump A : 0.1%trifluoroacetic in 100%water
Pump B : 0.1%trifluoroacetic in 100%acetonrtrile
Total Flow : 1.0ml/min
Wavelength : 214nm
Analytical column type : SHIMADZU shim-pack GIST(4.6*250MM*5UM)
Dissolution method : 0.1mg sample dissolved to 0.5mL by 20%ACN and 80%H2O
Acquisition Time : 2025/04/08 12:28:35
Inj.Volume : 30ul

Time	Module	Action	Value
0.01	Pumps	B.Conc	22
20.00	Pumps	B.Conc	42

Chromatogram

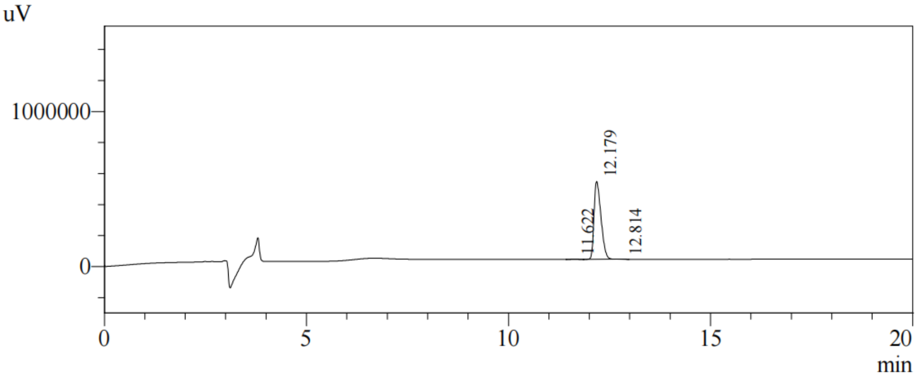

1 Det.A Ch1 / 214nm

PeakTable

Detector A Ch1 214nm

Peak#	Ret. Time	Area	Height	Area %	Height %
1	11.622	14893	1322	0.247	0.262
2	12.179	6003756	501759	99.647	99.585
3	12.814	6396	767	0.106	0.152
Total		6025045	503849	100.000	100.000

Figure S5. The chromatogram and the purity of the bioactive peptide sample SLFLPK.

Sample Information

Name	:	P36697-6
Sequence	:	DPFDALPK
Modification	:	N/A
Lot.No	:	P36697-6-25040201
Pump A	:	0.1%trifluoroacetic in 100%water
Pump B	:	0.1%trifluoroacetic in 100%acetonrtrile
Total Flow	:	1.0ml/min
Wavelength	:	214nm
Analytical column type	:	SHIMADZU shim-pack GIST(4.6*250MM*5UM)
Dissolution method	:	0.1mg sample dissolved to 0.5mL by 20%ACN and 80%H2O
Acquisition Time	:	2025/04/07 11:56:35
Inj.Volume	:	30ul

Time	Module	Action	Value
0.01	Pumps	B.Conc	21
20.00	Pumps	B.Conc	41

Chromatogram

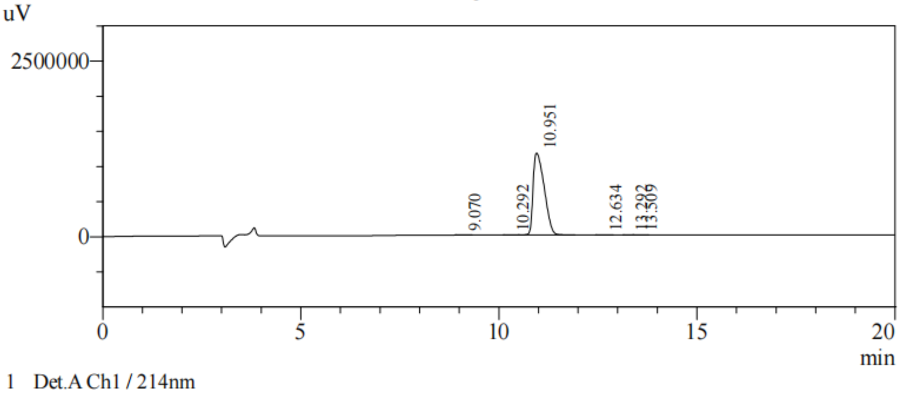

1 Det.A Ch1 / 214nm

PeakTable

Detector A Ch1 214nm

Peak#	Ret. Time	Area	Height	Area %	Height %
1	9.070	39606	3320	0.174	0.283
2	10.292	8447	596	0.037	0.051
3	10.951	22693262	1166050	99.676	99.458
4	12.634	8313	731	0.037	0.062
5	13.292	5145	522	0.023	0.045
6	13.509	12275	1184	0.054	0.101
Total		22767048	1172402	100.000	100.000

Figure S6. The chromatogram and the purity of the bioactive peptide sample DPFDALPK.

Table S1. The molecular docking results of bioactive peptides from yellow mealworm larvae with α-glucosidase.

Peptide sequences	DK-7	WK-6	GR-7	FK-8	SK-6	DK-8	WLRL (Positive control)
Affinity (kcal/mol)	-7.3	-8.2	-8.0	-8.0	-8.1	-7.9	-8.2
Number of hydrogen bonds	11	7	8	11	12	13	10
Average bond distances (Å)	3.4	3.4	3.3	3.4	3.2	3.4	3.4
Amino acid residues involved in hydrogen bonds	Asn14 Asp18 Gln19 Ser40 Thr196 Arg254 Thr469 Arg471	Glu271 Arg647 Arg653 Gly732 Tyr733 Glu760	Glu658 Asp697 Arg712 Tyr733 Asp759 Glu763 Lys765	Gln275 Arg643 Arg647 Arg653 Val699 Gly731 Tyr733	Arg643 Arg647 Arg653 Glu658 Tyr733 Glu767	Gln275 Arg643 Arg647 Arg653 Glu661 Arg730 Tyr733	Arg647 Asp649 Arg653 His657 Tyr660 Tyr733
Number of hydrophobic interactions	5	6	7	9	8	4	6
Amino acid residues involved in hydrophobic	Asp18 Pro21 Trp194 Val244 Phe468	Tyr636 Thr639 Leu640 Pro676 Tyr733 Glu767	Thr269 Glu271 Tyr633 Tyr733 Lys765 Glu767	Gln272 Thr639 Leu640 Tyr660 Pro676 Ala698 Tyr733 Lys765 Glu767	Thr269 Glu271 Thr639 Leu640 Lys765 Glu767	Tyr636 Phe659 Pro676 Glu767	Glu271 Gln272 Thr639 Pro676 Lys765 Glu767

interactions							
Molecular docking 3D structure models	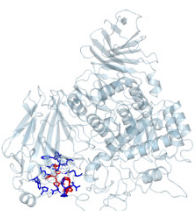	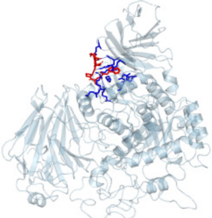	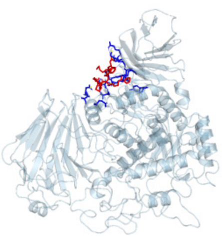	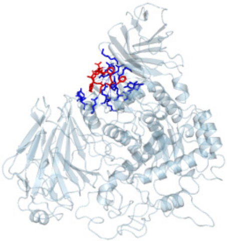	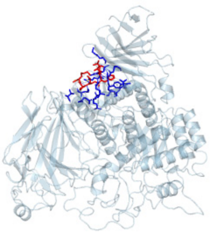	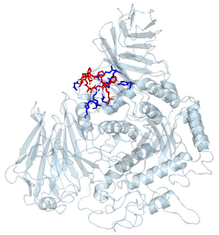	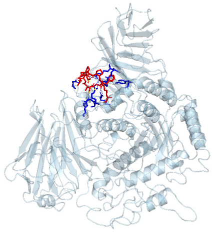

Table S2. The molecular docking results of bioactive peptides from yellow mealworm larvae with DPP-IV.

Peptide sequences	DK-7	WK-6	GR-7	FK-8	SK-6	DK-8	WLRL (Positive control)
Affinity (kcal/mol)	-8.4	-8.4	-9.0	-8.3	-7.8	-6.8	-7.6
Number of hydrogen bonds	11	9	9	12	12	11	7
Average bond distances (Å)	3.2	3.3	3.3	3.3	3.2	3.4	2.7
Amino acid residues involved in hydrogen bonds	Arg125 Glu205 Arg358 Tyr547 Gln553 Ser630 Tyr662 Asn710	Glu205 Glu206 Ser209 Arg356 Arg358 Glu361 Ile405 Glu408	Asp729 Gln731 Gln761	Tyr48 Arg125 Glu205 Val546 Tyr547 Asn562 Trp629 Gly632 Asn710 Tyr752	Arg125 Tyr547 Lys554 Asn562 Trp629 Ser630 Tyr662 Asn710 His740	Gln275 Arg643 Arg647 Arg653 Glu661 Arg730	Asp729 Gln731 His754 Gln761
Number of hydrophobic interactions	7	11	7	18	3	3	6
Amino acid residues involved in hydrophobic interactions	Phe357 Tyr547 Lys554 Trp629	Arg356 Phe357 Arg358 Ile405 Ile418 Arg429 Tyr666	Glu699 Gln731 Phe758 Gln761	Tyr547 Lys554 Asn562 Trp563 Ala564 Trp627 Trp629 Tyr631 Val656 Trp659 Tyr662 Tyr666 Tyr752	Tyr547 Lys554 Trp627	Glu271 Pro676 Glu767	Glu699 Gln731 His757 Phe758 Gln761

Molecular docking 3D structure models	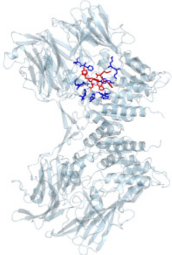	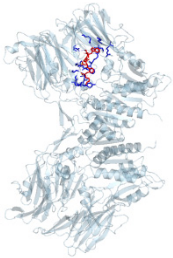	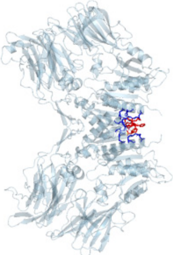	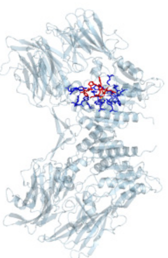	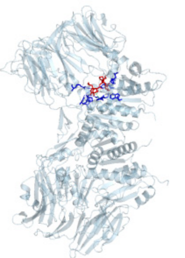	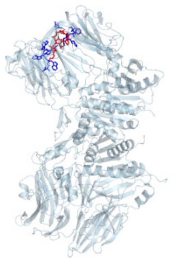	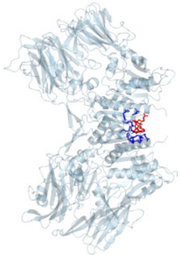
